# Urinary Outcomes Following a Novel Reconstructive Pelvic Organ Prolapse Surgery: Randomized Controlled Trial

**DOI:** 10.3390/medicina62040619

**Published:** 2026-03-25

**Authors:** Kristians Šušpanovs, Igors Ivanovs, Vilnis Lietuvietis, Ronalds Mačuks, Ieva Siksaliete, Dmitrijs Aleksandrovs, Dainis Krieviņš

**Affiliations:** 1 Women’s Health Center, Pauls Stradiņš Clinical University Hospital, 13 Pilsonu Str., LV-1002 Riga, Latvia; 2 Faculty of Medicine and Life Sciences, University of Latvia, 3 Jelgavas Str., LV-1004 Riga, Latvia; 3 Department of Emergency and General Surgery, Riga East Clinical University Hospital, Gaiļezers, 2 Hipokrata Str., LV-1079 Riga, Latvia; 4 Clinic of Urology and Oncological Urology, Riga East Clinical University Hospital, Gaiļezers, 2 Hipokrata Str., LV-1079 Riga, Latvia; 5 Faculty of Medicine, Rīga Stradiņš University Latvia, 16 Dzirciema Str., LV-1004 Riga, Latvia; 6 Department of Vascular Surgery, Pauls Stradiņš Clinical University Hospital, 13 Pilsonu Str., LV-1002 Riga, Latvia

**Keywords:** pelvic organ prolapse, urinary incontinence, de novo urinary incontinence, sacrocervicopexy, sacrocolpopexy, colporrhaphy

## Abstract

*Background and Objectives:* The close anatomical relationship between pelvic support structures and the lower urinary tract contributes to high rates of urinary dysfunction among patients with pelvic organ prolapse (POP). POP reduction alone has been shown to alter urinary tract functioning. The aim of this study was to assess urinary functioning outcomes following a novel reconstructive surgical technique for POP. *Materials and Methods*: This randomized controlled trial was conducted between September 2024 and December 2025. The trial was registered in the German Clinical Trials Register (identifier: DRKS00038206), on 27 October 2025. Participants were randomly assigned to undergo either conventional laparoscopic sacrocervicopexy or the modified technique. Urinary outcomes were assessed using the International Consultation on Incontinence Questionnaire—Urinary Incontinence Short Form (ICIQ-UI), the Urogenital Distress Inventory Short Form (UDI-6), the cough test, and urodynamic testing. Assessments were performed prior to surgery and at a 6-month follow-up. *Results*: Both the classical and modified techniques resulted in significant improvements in ICIQ-UI and UDI-6 scores. However, no statistically significant differences were observed between groups. De novo SUI occurred in 14.3% of patients in the classical technique group and in no patients in the modified technique group. *Conclusions*: Urinary symptoms improved in both groups, with no statistically significant between-group differences. De novo SUI occurred only in the classical technique group, but this finding should be interpreted cautiously given the limited sample size. These results are exploratory and hypothesis-generating, and larger studies with longer follow-up are needed to clarify whether true between-group differences in postoperative continence outcomes exist.

## 1. Introduction

The prevalence of POP varies worldwide, reaching 65% in some female populations [1]. It is associated with a wide range of symptoms involving bowel, sexual, and urinary function. Urinary tract symptoms are particularly common, affecting up to 88% of symptomatic women [2], and include urinary incontinence, abnormalities of bladder storage and sensation, and recurrent urinary tract infections [3]. These symptoms have a substantial negative impact on quality of life [4] and, in severe cases, can lead to life-threatening complications such as hydronephrosis, kidney failure [5], and urosepsis [6,7].

The relationship between POP and urinary symptoms is thought to reflect the close anatomical and functional integration of pelvic support structures with the lower urinary tract. The “hammock hypothesis” conceptualizes urethral support as a composite layer formed by the anterior vaginal wall and endopelvic fascia, stabilized laterally by attachments to the arcus tendineus fascia pelvis and the levator ani complex [8,9]. Consistent with this concept, prolapse reduction alone can improve urinary symptoms in a substantial proportion of women, even in the absence of concomitant anti-incontinence procedures [10,11]. Conversely, anatomical correction may unmask previously occult stress urinary incontinence or lead to de novo SUI [12].

Surgical management of POP can be broadly categorized into obliterative procedures and reconstructive procedures that preserve the vaginal vault [13]. Among reconstructive approaches, sacrocolpopexy (SCP) and sacrocervicopexy (SCerP) are among the most extensively studied techniques for prolapse repair [14,15,16]. The primary objective of both procedures is restoration of apical vaginal support by suspending the vaginal apex or uterine cervix to the anterior longitudinal ligament of the sacrum using a graft material [13]. Laparoscopic SCP and SCerP have demonstrated high and longstanding anatomical success, with objective success rates between 67% and 99% at follow-up [16]. Importantly, laparoscopic SCP performed without concomitant anti-incontinence surgery has been associated with significant improvements in voiding dysfunction [17], urinary incontinence [11,18], and symptoms of overactive bladder [11,19]. Nevertheless, despite successful anatomical restoration, de novo urinary symptoms—most commonly de novo SUI—may develop postoperatively [20]. Reported incidence rates vary widely, ranging from no cases in some studies [11] to up to 27% in others [21]. Identified risk factors include a higher body mass index, preoperative urinary urgency, prior transvaginal mesh surgery for POP, and potentially surgical technique-related factors [18,22].

In addition to apical suspension procedures, reconstructive management of POP may include vaginal wall repair techniques such as colporrhaphy. In cases of posterior compartment prolapse, colporrhaphy—with or without levator ani muscle plication—may be performed to restore posterior vaginal wall support [13]. Posterior compartment surgery has primarily been studied in the context of bowel and sexual function [23]. Although Glavind and Christiansen reported improvements in urinary incontinence in selected patient groups after posterior repair [24], comparative evidence linking posterior reconstruction strategies to patient-reported urinary symptom burden and objective continence testing is limited. This represents an important clinical gap, as combined apical and posterior repairs are commonly performed in women with multicompartment prolapse, yet their potential influence on postoperative urinary outcomes is not routinely quantified [25]. The modified technique evaluated in this study differs from conventional SCerP practices [13] by incorporating distal graft anchoring to the uterosacral ligaments in accordance with the McCall technique [3] and posterior colporrhaphy with levator ani plication. The primary aim of integrating these procedures was to increase the number of anatomical fixation points, thereby reinforcing apical and posterior compartment support. However, the additional fixation and posterior reconstruction may alter pelvic floor geometry and the vaginal axis, with potential implications for bladder neck position, urethral mobility, and postoperative lower urinary tract symptoms. We hypothesized that the modified approach could be associated with a different postoperative urinary symptom trajectory compared with conventional laparoscopic SCerP. As the modified technique includes posterior vaginal wall repair, analysis of urinary outcomes may provide further insight into the functional effects of this procedure and support technique selection in the surgical management of POP. Therefore, the aim of this study was to compare urinary outcomes between the conventional and modified techniques.

## 2. Materials and Methods

This prospective randomized controlled clinical trial was conducted between September 2024 and December 2025 at Pauls Stradiņš Clinical University Hospital and Riga East Clinical University Hospital, Riga, Latvia. The study protocol was reviewed and approved by the Riga East Clinical University Hospital Ethics Committee on Medical and Biomedical Research (approval No. 16-A/24) on 29 August 2024, prior to participant enrollment.

The trial was retrospectively registered in public clinical trial registries. Registration in the German Clinical Trials Register, BfArM.de (identifier: DRKS00038206), was completed on 27 October 2025, followed by registration in the United States National Library of Medicine registry, ClinicalTrials.gov (identifier: NCT07271862), on 6 December 2025.

**Figure 1:**
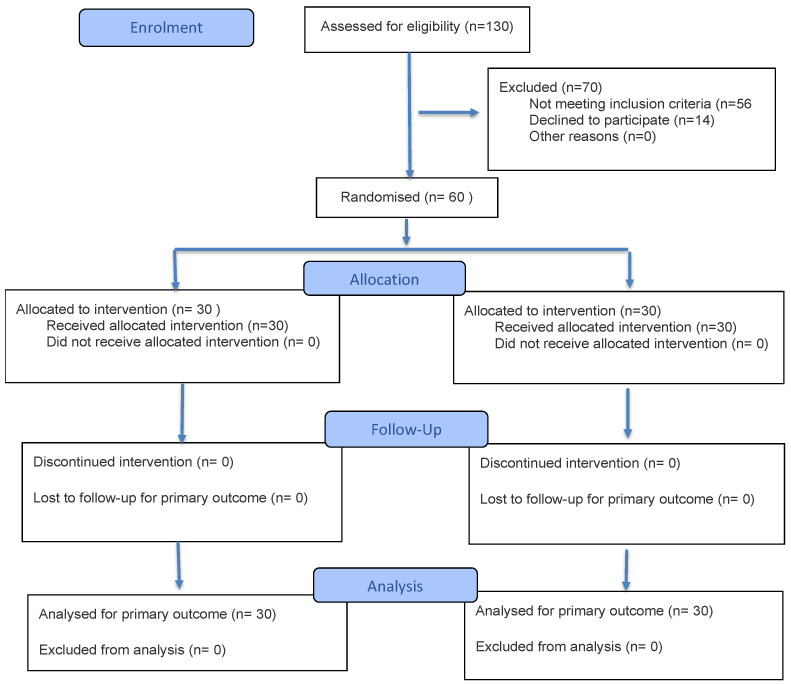
Study flow diagram in accordance with CONSORT 2025.

This study was initiated as a local health care service improvement project within the participating institutions. In accordance with local institutional policy, formal ethics committee approval—including full protocol and statistical analysis plan review—was required before initiation. At the time the study commenced, ethics approval was considered the primary regulatory prerequisite at the institutional level. While conducting the study, a decision was made to disseminate the findings to clinical practices beyond the participating centers. Therefore, the trial was registered retrospectively to ensure public accessibility of the protocol and prespecified analyses in line with CONSORT recommendations [26]. The study was registered in a second registry to ensure timely public posting. The trial was conducted and is reported in accordance with the CONSORT 2025 guidelines (Figure 1) [26]. The full study protocol is publicly available through the trial registries.

As defined in the parent trial protocol, which is publicly available in the trial registry, the primary outcome was anatomical correction of pelvic organ prolapse, assessed according to changes in Pelvic Organ Prolapse Quantification (POP-Q) measurements. Secondary outcomes included pelvic floor symptoms, sexual function, urinary function, intraoperative and postoperative complications, operative time, length of hospital stay, and quality of life.

This manuscript reports a secondary analysis focused on urinary outcomes, including urodynamic findings, cough test results, ICIQ-UI, and UDI-6. These outcomes were specified in the study protocol before enrollment of the first participant and were not selected post hoc. Pelvic floor symptoms were assessed using the PFDI-20, including its UDI-6 subscale, and urinary function was evaluated using the cough test, ICIQ-UI, and urodynamic testing. Urinary outcomes were the focus of this analysis; though they were not considered primary endpoints, POP-Q measurements are reported to describe baseline prolapse severity and the anatomical context of the surgical indication.

### 2.1. Study Participants

The participants were divided into two groups of 30 patients each. The first group underwent conventional laparoscopic SCerP [12] and was defined as the classical technique group. The second group underwent the modified technique. No formal study power calculation was performed. The target sample size (*N* = 60) was determined based on feasibility at the participating centers within the recruitment period.

Inclusion criteria were POP of stage 2 or greater according to POP-Q classification [27], presence of posterior vaginal wall prolapse (defined as prolapse involving POP-Q points Ap and/or Bp), age between 30 and 80 years, presence of a retained cervix (either with the uterus in situ or after previous supracervical hysterectomy), and no prior surgical treatment for POP or urinary incontinence. Exclusion criteria included severe extragenital pathology contraindicating surgery, a history of malignant pelvic disease, and a lack of POP-related symptoms. POP-related symptoms were defined as the presence of a vaginal bulging sensation and/or urinary, sexual, or anorectal symptoms.

Participants were randomized at a 1:1 ratio to the classical technique group or the modified technique group using a computer-generated random sequence. Randomization was performed with permuted blocks of variable size to maintain balance between groups. The allocation sequence was generated by a study team member who was not involved in participant recruitment, clinical assessment, or surgery. Allocation concealment was ensured using sequentially numbered, opaque, sealed envelopes prepared in advance according to the randomization list. After confirming eligibility and informed consent, the enrolling investigator opened the envelopes in sequence to reveal group assignment.

### 2.2. Data Collection

Participants were evaluated preoperatively and at a 6-month follow-up. Assessments included urodynamic testing, a questionnaire on demographic characteristics and medical history, the ICIQ-UI, and the UDI-6. Latvian translations of both questionnaires were used. Both tools are validated and widely used in clinical practice and research for pelvic floor disorders [28]. The ICIQ-UI is used to evaluate urinary incontinence and its impact on quality of life [29]. ICIQ-UI scores range from 0 to 21, with higher scores indicating greater symptom severity. The UDI-6 is used to evaluate the presence of lower urinary tract symptoms and the degree of inconvenience they cause the patient [30]. UDI-6 scores were calculated on the standard transformed 0–100 scale, with higher scores indicating greater symptom burden. Assessment with the cough test was also performed [31]. POP severity was evaluated using the POP-Q classification system.

Urodynamic testing was performed using a standardized protocol comprising filling cystometry with coughing as a provocative maneuver and pressure–flow studies. Testing was conducted in a sitting position. Filling cystometry was performed using saline at a filling rate of 50 mL/min via a double-lumen transurethral catheter. Urodynamic stress incontinence was defined as involuntary leakage during filling cystometry associated with increased intra-abdominal pressure in the absence of a detrusor contraction. Leak point pressure was recorded as the lowest vesical pressure at which leakage occurred during provocation in the absence of detrusor contraction (ALPP/VLPP). For descriptive analyses, SUI was stratified by leak point pressure into three severity categories: severe (<60 cmH_2_O), moderate (60–90 cmH_2_O), and mild (>90 cmH_2_O). The SUI captured during urodynamic testing was used as the SUI definition during data analysis.

Occult SUI was assessed in all participants during the preoperative urodynamic evaluation by performing prolapse reduction using a vaginal speculum during filling cystometry. Occult SUI was defined as urine leakage observed only after prolapse reduction, with no leakage prior to reduction. During analysis, occult SUI was classified as having SUI.

SUI resolution was defined as the absence of urine leakage during postoperative filling cystometry among participants who demonstrated SUI on the preoperative urodynamic assessment, including those with occult SUI. De novo SUI was defined as urine leakage during postoperative filling cystometry among participants without preoperative SUI. Thus, the denominator for resolution and persistence outcomes comprised participants with baseline SUI, whereas the denominator for de novo SUI comprised participants without baseline SUI. The postoperative follow-up urodynamic test and interpretation were performed by a urogynecology specialist who was blinded to the surgery group division.

### 2.3. Surgical Technique

In the classical technique group, SCerP was performed using a polypropylene mesh implant. The distal portion of the mesh was secured to the cervix and along the full length of the anterior vaginal wall up to the level of the bladder neck. Additional fixation was applied along the posterior vaginal wall and to the levator ani muscles. Physiological tension was ensured, and the mesh was fixed proximally to the sacral promontory at the level of the anterior longitudinal ligament. Fixation was performed using separate intracorporeal nonabsorbable sutures placed at 1.5–2.0 cm intervals. No vaginal repair was performed.

The modified procedure consisted of laparoscopic SCerP combined with vaginal wall reconstruction using native tissues. A polypropylene graft was placed with distal fixation to the bilateral uterosacral ligaments according to McCall’s technique [3], to the cervix, and along the anterior vaginal wall up to the bladder neck, maintaining physiological tension. Proximal fixation to the anterior longitudinal ligament was performed. During the laparoscopic SCerP, separate intracorporeal nonabsorbable sutures were placed 1.5–2.0 cm apart. Posterior colporrhaphy with simultaneous levator ani plication was subsequently performed via the vaginal route, also known as vaginal colpoperineolevatoroplasty.

All procedures were performed by an attending surgeon who was blinded to the preoperative questionnaire and urodynamic results.

### 2.4. Statistical Analysis

Continuous quantitative measures were summarized as medians (interquartile range IQR: Q1–Q3), and categorical variables were summarized as counts and percentages. For baseline participant demographic and anthropometric characteristics, balance was assessed using standardized mean differences (SMDs). Normality for continuous variables was evaluated using the Shapiro–Wilk test and, given the observed non-Gaussian distribution and the small sample size, non-parametric tests were used for further analyses. Within-group pre-to-post differences in continuous and ordinal outcomes were assessed using the Wilcoxon signed-rank test, and between-group comparisons at each time point were performed using the Mann–Whitney U test.

For multi-category nominal outcomes measured pre- and post-intervention, within-group changes were assessed using the Stuart–Maxwell test (paired marginal homogeneity), with Bowker’s test of symmetry used as a complementary assessment of paired transition symmetry. Between-group comparisons of category distributions at each time point were performed using exact or simulation-based extensions of Fisher’s test for R × C tables (Fisher–Freeman–Halton/Fisher with Monte Carlo simulation) where sparse expected counts made asymptotic Chi-square methods inappropriate.

In case of longitudinal comparisons of questionnaire scores between time points and between techniques, repeated-measures models were used with fixed effects for technique, time, and the Technique × Time interaction, and participant-level correlation was accounted for using a subject-specific intercept (mixed-effects framework) or a marginal correlation structure (generalized estimating equation framework), depending on the outcome/model implementation. Models for questionnaire scores were specified with a Gaussian distribution and identity link and included fixed effects for Technique, Time, and the Technique × Time interaction. No covariates were included.

For questionnaire outcomes analyzed with repeated-measures models, we reported estimated marginal means (EMMs) and prespecified contrasts to obtain (i) between-group differences at baseline and at follow-up, (ii) within-group pre-to-post changes in each technique group, and (iii) the Technique × Time interaction (between-group difference in change). Multiplicity adjustment was performed using the Holm step-down procedure. Unadjusted *p*-values are reported as Punadj and multiplicity-adjusted *p*-values as Padj.

For Binary paired outcomes, we used the McNemar (exact when sparse). For between-group categorical comparisons, we used the Fisher/Fisher–Freeman–Halton/Monte Carlo when sparse. Binary and categorical outcome results were summarized using effect estimates with 95% confidence intervals. For low-frequency binary outcomes, exact (Clopper–Pearson) 95% confidence intervals are reported for proportions. For risk differences (differences in proportions between groups), 95% confidence intervals were computed using the Newcombe method (without continuity correction). For model-based estimates from Gaussian identity-link repeated-measures models (e.g., estimated changes, between-group contrasts, and Technique × Time interaction estimates), 95% confidence intervals were computed using Wald methods (estimate ± 1.96 × standard error). For GEE logistic models, 95% confidence intervals for odds ratios were computed using robust standard errors on the log-odds scale and exponentiated.

Model adequacy was assessed by confirming successful model convergence without warnings. For Gaussian identity-link repeated-measures models, residual diagnostics were reviewed to check for gross departures from model assumptions. For generalized estimating equation (GEE) models, inference was based on robust (sandwich) standard errors, and sensitivity to the working correlation structure was evaluated by refitting the primary models under alternative structures (independence, exchangeable, AR(1), and unstructured). The primary Technique × Time estimates were unchanged across correlation structures (e.g., for the binary urodynamic SUI model, Technique × Time OR ≈ 0.51 with 95% CI ≈ 0.13–1.98 under all structures).

All tests were two-sided, and statistical significance was assessed at α = 0.05. Analyses were performed according to the intention-to-treat principle, with participants analyzed in the groups to which they were randomized. All statistical analysis and visualizations were performed using IBM SPSS Statistics v30.0 (IBM Corp., Chicago, IL, USA) and R v4.5.1, accessed via RStudio.

## 3. Results

Across the two study groups, baseline demographics and anthropometric measures were observed to be generally comparable. Some imbalances were observed. Infant birthweight and number of comorbidities had SMDs of less than 0.10, indicating balance. Moderate imbalances were observed in height, diabetes count, body mass index (BMI), age, and hysterectomy history, with SMDs ranging from 0.10 to 0.30. The greatest imbalances were seen in weight, chronic obstructive pulmonary disease (COPD) count, and time since POP diagnosis, with SMDs above 0.30. Parity had wide-ranging balance, as indicated by SMD. Counts for parities of II and III were well-balanced; however, parity of IV had a moderate SMD value, and parities of I and V had large SMD values. There were no missing outcome data at the baseline and 6-month assessments (Table 1).

**Figure 2:**
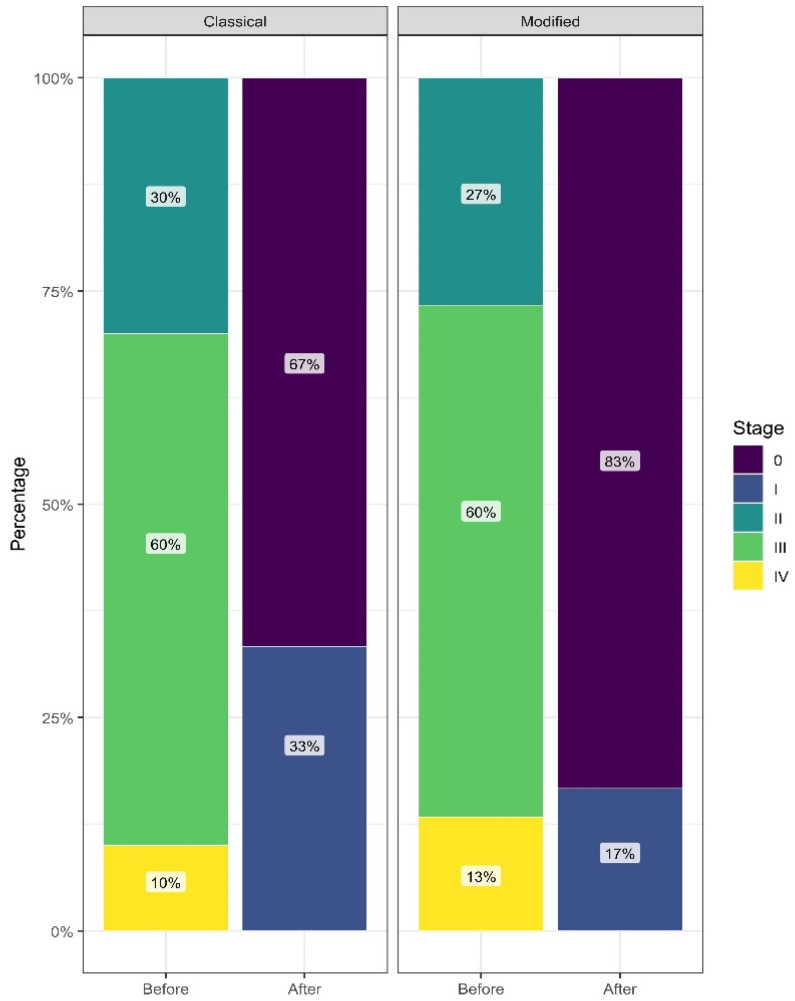
Distribution of the POP-Q stage before and after surgery by technique group.

Preoperatively, POP-Q point values were broadly comparable between the classical and modified technique groups across all those assessed, with no significant compartment differences. Postoperatively, both groups demonstrated a marked shift toward lower stages, with participants classified predominantly as stage 0 or stage I. Postoperative stage distributions did not differ significantly between groups (Mann–Whitney U, *p* = 0.142). Within-group paired analyses indicated significant improvement in stage from pre- to post-assessment in both groups (Wilcoxon signed-rank, classical *p* < 0.0001; modified *p* < 0.0001). The descriptive results are summarized in Figure 2.

Both technique groups showed statistically significant reductions in UDI-6 scores when comparing postoperative and preoperative results. The median (IQR) UDI-6 was 47.5 (32.0 to 56.0) at baseline and 7.0 (0.0 to 8.0) postoperatively in the classical technique group and 45.8 (27.8 to 59.5) at baseline and 1.0 (0.0 to 2.2) postoperatively in the modified technique group. There was no statistically significant between-group difference at baseline median values (Mann–Whitney U test: *p* = 0.859). The estimated change was −35.29 (95% CI: −42.14 to −28.43; Punadj < 0.0001; Padj < 0.0001) in the classical technique group and −41.48 (95% CI: −48.34 to −34.62; Punadj < 0.0001; Padj < 0.0001) in the modified technique group. Between-group comparisons did not demonstrate significant differences at baseline (estimate 1.63, 95% CI: −5.62 to 8.89; Punadj = 0.657; Padj = 1.000) or postoperatively (estimate −4.56, 95% CI: −11.81 to 2.69; Punadj = 0.216; Padj = 0.618). The Technique × Time interaction (difference in pre-to-post change between techniques) was not statistically significant (estimate −6.19, 95% CI: −15.89 to 3.50; Punadj = 0.206; Padj = 0.618). Categorized results are presented in Table 2.

Preoperatively, ICIQ-UI scores were similar between the classical and modified technique groups (Table 3). Median (IQR) ICIQ-UI was 7.0 (4.0 to 12.0) in the classical group and 6.5 (3.0 to 12.0) in the modified group. The GEE model baseline contrast was not statistically significant (estimate −0.27, 95% CI: −2.28 to 1.75; Punadj = 0.795; Padj = 1.000). Postoperatively, ICIQ-UI scores decreased in both groups. The estimated change was −6.40 (95% CI: −7.70 to −5.10; Punadj < 0.0001; Padj < 0.0001) in the classical technique group and −6.47 (95% CI: −8.69 to −4.24; Punadj < 0.0001; Padj < 0.0001) in the modified technique group. Between-group comparisons did not demonstrate significant differences postoperatively (estimate −0.33, 95% CI: −2.35 to 1.68; Punadj = 0.745; Padj = 1.000). The repeated-measures model including a Technique × Time interaction showed no evidence of differential change by technique (estimate −0.07, 95% CI: −2.75 to 2.62; Punadj = 0.960; Padj = 1.000), indicating that the magnitude of pre-to-post improvement did not differ between groups.

Baseline urodynamic test category distributions did not differ between groups (Fisher’s exact test with Monte Carlo simulation, *p* = 0.758). Postoperatively, the distribution did not differ significantly between groups (Fisher’s exact test with Monte Carlo simulation, *p* = 0.310). Within-group paired analyses did not show statistically significant pre-to-post changes in the marginal distribution in either group (classical technique: Stuart–Maxwell χ^2^ = 5.00, df = 4, *p* = 0.287; modified technique: Stuart–Maxwell χ^2^ = 7.00, df = 4, *p* = 0.136). Similarly, Bowker’s symmetry test did not indicate statistically significant asymmetry in the paired transition tables for either group (classical technique: χ^2^ = 5.00, df = 2, *p* = 0.082; modified technique: χ^2^ = 7.00, df = 3, *p* = 0.072). As shown in Table 4, urodynamic SUI of any type was present at the 6-month follow-up in 7/30 (23.3%) patients in the classical technique group and 3/30 (10.0%) in the modified technique group, corresponding to an absolute risk difference (modified − classical) of −13.3 percentage points (95% CI: −32.1 to 6.1). To directly assess whether change over time differed by technique, we fitted a longitudinal GEE logistic model for the binary urodynamic endpoint (any SUI versus none). The Technique × Time interaction was not statistically significant (OR 0.51, 95% CI: 0.13 to 1.98; *p* = 0.333).

SUI was resolved in five out of nine patients (55.6%, 95% CI: 21.2% to 86.3%) in the classical technique group and in four out of seven patients (57.1%, 95% CI: 18.4% to 90.1%) in the modified technique group, corresponding to an absolute risk difference of 1.6 percentage points (95% CI: −56.1 to 57.5, Fisher’s exact test, *p* = 1.000). De novo SUI was observed in 3 out of 21 patients (14.3%, 95% CI: 3.0% to 36.3%) in the classical technique group, whereas no cases were identified among the 23 patients in the modified technique group who were at risk of de novo SUI (0%, 95% CI: 0.0% to 14.8%), corresponding to an absolute risk difference of −14.3 percentage points (95% CI: −34.6 to 9.3). However, the between-group comparison for de novo SUI was not statistically significant (Fisher’s exact test, *p* = 0.100).

At baseline, the distribution of cough test categories (positive, negative, and equivocal) did not differ between groups (Fisher–Freeman–Halton test, *p* = 0.776). Postoperatively, the distribution remained similar between groups, with a borderline between-group difference that did not reach statistical significance (Fisher–Freeman–Halton test, *p* = 0.085). Notably, the positive category was not observed postoperatively in either group. Within-group paired analyses demonstrated no statistically significant change in the marginal distribution of cough test categories from pre to post assessment in the classical technique group (Stuart–Maxwell χ^2^ = 4.17, df = 2, *p* = 0.125; Bowker symmetry test *p* = 0.241). In contrast, the modified technique group demonstrated a statistically significant shift towards improvement in cough test category distribution over time (Stuart–Maxwell *p* = 0.025; Bowker *p* = 0.046).

## 4. Discussion

One of the key observations of this study is the meaningful improvement in urinary symptoms despite the absence of concomitant anti-incontinence surgery. Across the patient-reported outcomes (PROMs), both techniques were associated with a substantial improvement from baseline to 6 months. Both techniques also achieved the primary surgical objective of prolapse correction, with a significant improvement in POP-Q measurements. The anatomical results were within the range reported in previous SCP studies [16].

To capture both objective and subjective aspects of urinary function, we used urodynamic testing, a clinician-guided cough test, and patient-reported outcome measures. Urodynamic testing was used to identify and classify SUI, including preoperative occult SUI, whereas the cough test and PROMs captured clinically observable leakage and symptom burden.

In both groups, ICIQ-UI and UDI-6 scores improved significantly from baseline. However, no statistically significant between-group differences were observed, and model-based longitudinal analyses did not demonstrate differential pre-to-post change by technique for either questionnaire. No participant in either group had a positive postoperative cough test. Although the modified technique group showed a significant within-group improvement in cough test category distribution over time, the postoperative between-group comparison did not reach statistical significance. The results suggest an overall improvement in urinary outcomes in both groups without clear evidence of a differential effect between techniques.

The urodynamic SUI results were classified into severity categories to facilitate interpretation alongside changes in ICIQ-UI, UDI-6, and cough test findings. No statistically significant between-group difference was observed in postoperative urodynamic category distribution. The urodynamic findings were interpreted in the context of the PROMs, which indicated an overall improvement in urinary symptoms. This underscores the importance of combining objective and patient-reported measures of urinary function in clinical care. Previous studies have also reported an improvement in urinary function after prolapse correction without concomitant anti-incontinence surgery. Urodynamic SUI resolution was observed in the majority of affected patients in both groups: 55.6% in the classical technique group and 57.1% in the modified technique group. These rates fall within the ranges reported in previous studies on reconstructive POP surgery. In a nationwide study, 39% of women reported complete SUI resolution 6 months after POP surgery alone [32]. Illiano et al. reported resolution of SUI in approximately 65% of women after SCP alone [12], while Christmann-Schmid et al. observed resolution in 69% of patients [19].

POP surgery may be associated with de novo SUI [16]. In the present trial, de novo SUI occurred in 14.3% of patients undergoing the classical technique and in none of the patients undergoing the modified procedure. However, this between-group difference was not statistically significant. The observed incidence in the classical group is consistent with previously reported rates after SCP, including reports of no cases in some series [12] and incidences of 13% [33] and 17% [23] in others.

One proposed mechanism of de novo SUI after prolapse correction is unmasking of stress leakage when prolapse-related urethral kinking or functional outlet obstruction is relieved. However, this mechanism alone is unlikely to explain all cases, as de novo SUI has also been reported in women without occult SUI on preoperative testing [34]. This suggests that postoperative stress leakage may also reflect changes in outlet mechanics, bladder neck support, or periurethral tissue after reconstructive surgery [35]. In our cohort, no cases of de novo SUI were observed in the modified technique group. This finding may suggest that the additional or redistributed support points of the modified technique—distal graft anchoring to the uterosacral ligaments together with posterior colporrhaphy and levator ani plication—could influence postoperative support vectors and outlet mechanics. However, given the limited sample size and event counts, this observation should be interpreted cautiously and as hypothesis-generating rather than as evidence of a definitive protective effect.

A primary methodological limitation of this study is the retrospective timing of trial registration. Although the registration details are transparently reported and urinary outcomes were defined in the original protocol before participant enrollment, retrospective registration remains an important limitation that should be considered when interpreting the findings.

Although randomization was used, some baseline characteristics showed a moderate to high imbalance between groups, particularly weight, COPD prevalence, time since POP diagnosis, and parity distribution. From a clinical perspective, these factors—particularly higher body weight and chronic cough associated with COPD—may increase intra-abdominal pressure and thereby influence the incidence or severity of SUI. While these imbalances did not lead to significant differences in baseline symptom scores or urodynamic results, their potential influence on postoperative urinary outcomes cannot be excluded.

Furthermore, another limitation is the 6-month follow-up period. While this interval is sufficient to assess short-term functional outcomes, it may not capture late-onset urinary symptoms or the longer-term durability of urinary outcomes after prolapse reconstruction. Follow-up duration varies substantially across studies [2]. The existing literature suggests that urinary symptoms may continue to evolve beyond the first 6 months. For instance, Karjalainen et al. observed that SUI resolution rates declined from 39% to 35% between 6 and 24 months, while the incidence of de novo SUI symptoms rose from 15% to 20% [32]. Similarly, the CARE trial demonstrated a progressive increase in stress incontinence composite rates from 40.9% at 3 months, to 41.6% at 12 months, and 45.2% at 24 months [36]. Aichner et al. reported a similar longitudinal progression, with SUI rates increasing from 23.1% at 3 months to 27.7% at a mean follow-up of 20.3 months [17]. These data suggest that both SUI resolution and de novo SUI rates may change over time, indicating that 6-month estimates may not fully reflect longer-term continence outcomes.

Interpretation of the findings was limited by the relatively small sample size, which reduced statistical power. This is reflected in the wide 95% confidence intervals, particularly for low-frequency outcomes such as de novo SUI and SUI resolution. With its sample size, this study had adequate power to detect only relatively large between-group effects—a standardized mean difference of about 0.74 for continuous outcomes or absolute between-group differences of roughly 30–35 percentage points for binary outcomes, depending on the baseline event rate. Accordingly, non-significant between-group findings should not be interpreted as evidence of equivalence, and clinically important differences in low-frequency outcomes cannot be excluded. The urinary analyses should, therefore, be interpreted cautiously as exploratory. A follow-up study with a formal sample size calculation is warranted.

## 5. Conclusions

In this study, urinary symptoms improved substantially in both groups, as reflected by significant reductions in ICIQ-UI and UDI-6 scores. However, no statistically significant between-group differences were observed. De novo SUI occurred only in the classical technique group; although the study was underpowered for a definitive comparison, this pattern merits further investigation. Overall, these urinary findings should be interpreted as exploratory and hypothesis-generating. Larger studies with longer follow-up are needed to determine whether the observed patterns reflect true between-group differences in postoperative continence outcomes.

## Figures and Tables

**Table 1:** Baseline characteristics of the study participants.

Parameter	Classical Technique (*n* = 30)	Modified Technique (*n* = 30)	Difference *	|SMD|
Age (years)	59 (55 to 65)	59 (55 to 66)	−2.00	0.275
Weight (kg)	79 (70 to 84)	83 (69 to 98)	+5.40	0.349
Height (cm)	170 (167 to 175)	171 (161 to 186)	+1.80	0.169
BMI (kg/m^2^)	26 (23 to 29)	27 (23 to 33)	+1.39	0.261
Comorbidities (*n*, %)				
Diabetes mellitus	7 (23%)	5 (17%)	−6.7 pp	0.167
COPD	0 (0%)	2 (7%)	+6.7 pp	0.371
No comorbidity	23 (77%)	23 (77%)	0.0 pp	0.000
Parity (*n*, %)				
I	7 (23%)	3 (10%)	−13.3 pp	0.358
II	14 (47%)	14 (47%)	0.0 pp	0.000
III	7 (23%)	7 (23%)	0.0 pp	0.000
IV	2 (7%)	4 (13%)	+6.7 pp	0.222
V	0 (0%)	2 (7%)	+6.7 pp	0.371
Infant birthweight (*n*, %)				
≤4000 g	17 (57%)	16 (53%)	+3.3 pp	0.067
>4000 g	13 (43%)	14 (47%)		
Prior supracervical hysterectomy (*n*, %)				
Yes	7 (23%)	10 (33%)	+10.0 pp	0.222
No	23 (77%)	20 (67%)		
Time since POP diagnosis (months)	36 (30 to 51)	34 (21 to 45)	−7.87	0.485

SMD = standardized mean difference; pp = percentage points. Quantitative scale measures are presented as medians (Q1–Q3) and categorical variables as *n* (%). * for continuous variables, the difference value denotes the mean difference on the original scale; for categorical variables, it denotes the percentage-point difference.

**Table 2:** Categorical distribution of participants according to the UDI-6.

UDI-6 (*n*, %)	Classical Technique (*n* = 30)		Modified Technique (*n* = 30)	
	Before	After	Before	After
Without symptoms	2 (7%)	9 (30%)	0 (0%)	9 (30%)
Mild symptoms	7 (23%)	21 (70%)	11 (38%)	21 (70%)
Moderate symptoms	18 (60%)	0 (0%)	14 (45%)	0 (0%)
Severe symptoms	3 (10%)	0 (0%)	5 (17%)	0 (0%)

Severity cutoffs were as follows: 0 = without urinary symptoms; 1–33 = mild; 34–66 = moderate; 67–100 = severe.

**Table 3:** Categorical distribution of patients for ICIQ-UI scores.

ICIQ-UI Score (*n*, %) *	Classical Technique (*n* = 30)		Modified Technique (*n* = 30)	
	Before	After	Before	After
**Without**	0 (0%)	17 (57%)	0 (0%)	16 (53%)
**Mild**	14 (47%)	11 (37%)	14 (47%)	14 (47%)
**Moderate**	10 (33%)	2 (7%)	10 (33%)	0 (0%)
**Severe**	4 (13%)	0 (0%)	6 (20%)	0 (0%)
**Extreme**	2 (7%)	0 (0%)	0 (0%)	0 (0%)

* severity cutoffs: 0—without; 1 to 5—mild; 6 to 12—moderate; 13 to 18—severe; 19 to 21—extreme.

**Table 4:** Distribution of participants for results of urodynamic and cough tests.

Test	Classical Technique (*n* = 30)		Modified Technique (*n* = 30)	
	Before	After	Before	After
Urodynamic diagnoses of SUI (*n*, %)				
Without SUI	21 (70%)	23 (77%)	23 (77%)	27 (90%)
Mild	5 (17%)	7 (23%)	4 (13%)	3 (10%)
Moderate	0 (0%)	0 (0%)	1 (3%)	0 (0%)
Severe	0 (0%)	0 (0%)	0 (0%)	0 (0%)
Occult *	4 (13%)	N/A	2 (7%)	N/A
Cough test (*n*, %)				
Positive	4 (13%)	0 (0%)	5 (17%)	0 (0%)
Negative	24 (80%)	25 (83%)	22 (73%)	29 (97%)
Equivocal	2 (7%)	5 (17%)	3 (10%)	1 (3%)

N/A, not applicable. * occult SUI was assessed preoperatively only and was classified as SUI.

## Data Availability

Data are unavailable due to privacy restrictions.
